# Chestnut Species and Jasmonic Acid Treatment Influence Development and Community Interactions of Galls Produced by the Asian Chestnut Gall Wasp, *Dryocosmus kuriphilus*

**DOI:** 10.1673/031.011.14001

**Published:** 2011-10-20

**Authors:** William R. Cooper, Lynne K. Rieske

**Affiliations:** ^1^ University of Kentucky, Department of Entomology, S-225 Ag North, Lexington KY 40546-0091; ^2^ Current address: USDA-ARS, 17053 North Shafter Ave, Shafter, CA 93263

**Keywords:** chestnut blight, Cynipidae, endophyte, lesser chestnut weevil, plant signaling compounds, *Torymus*

## Abstract

Jasmonic acid (JA) is a plant—signaling hormone involved in defenses against insects and pathogens as well as the regulation of nutrient partitioning. Gall wasps (Hymenoptera: Cynipidae) induce the formation of galls on their host plants, which house immature wasps and provide them with nutrition and protection. The goal of this study was to investigate the effects of JA application on gall development and defenses. *Dryocosmus kuriphilus* Yasumatsu (Hymenoptera: Cynipidae) galls on American chestnut, *Castanea dentata* (Marsh.) Borkhausen (Fagales: Fagaceae), and Chinese chestnut, *C. mollissima* Blume, were treated with JA or a JA– inhibitor, diethyldithiocarbamic acid (DIECA), to determine the effects of these treatments on gall characteristics and defenses. Chinese chestnut galls treated with JA had greater volume and dry weight, thicker sclerenchyma layers, and fewer external fungal lesions compared with controls. Galls from both chestnut species treated with JA contained a lower proportion of empty chambers, and elevated tannin levels compared with controls. The effects of DIECA on galls were generally opposite from those of JA. American chestnut galls treated with DIECA had lower dry weight and fewer feeding punctures caused by the lesser chestnut weevil compared with controls. Galls from both chestnut species that were treated with DIECA were smaller and had more external fungal lesions compared with controls. Compared to American chestnut galls, Chinese chestnut galls had increased parasitism rates and fewer gall wasps. This study is the first to investigate the effects of JA on an insect gall, and indicates that JA treatments benefit gall wasps by increasing gall size and defenses.

## Introduction

*Dryocosmus kuriphilus* Yasumatsu (Hymenoptera: Cynipidae) is a globally important gall wasp that induces galls on actively growing shoots of all chestnut species (*Castanea* spp.). *Dryocosmus kuriphilus* galling prevents flower and shoot development, and can contribute to tree mortality. *Dryocosmus kuriphilus* is native to mainland Asia, but was accidently introduced in Japan, North America, and Europe, where it is a significant pest of cultivated chestnuts. *Dryocosmus kuriphilus* also threatens efforts to breed blight—resistant American chestnuts and introduce them to Appalachian forests (Anagnostakis 2001). In North America, *D. kuriphilus* is readily parasitized by hymenopteran parasitoids, and gall exteriors are damaged by the formation of lesions, thought to be induced by fungal endophytes and also by feeding from the lesser chestnut weevil, *Curculio sayi* (Cooper and Rieske 2007, 2009, 2010).

Insect galls, which are formed entirely from plant tissues, provide the gall inducers with refuge from natural enemies by providing physical barriers from predators, parasitoids, and pathogens (Cornell 1983; Price et al. 1987; Taper and Case 1987; Hartley and Lawton 1992; Cooper and Rieske 2010). In addition, the induction of galls establishes gall locations as active nutrient sinks, providing gall inducers with a continuous supply of nutrients (Hartley and Lawton 1992; Hartley 1998; Allison and Schultz 2005).

Physiological plant processes involved with plant defenses are regulated in part by the octadecanoid signal—transduction pathway (reviewed by Creelman and Mullet 1997; Halitschke and Baldwin 2005; Schilmiller and Howe 2005; Wasternack 2007). Jasmonates such as jasmonic acid (JA), methyl jasmonate, and JA—isoleucine are central signaling compounds of this pathway (Schaller 2001; Staswick and Tiryaki 2004). Jasmonate synthesis is triggered by physiological stresses, and can be artificially induced using foliar applications of synthetic jasmonates (reviewed by Creelman and Mullet 1997). A wide range of plant species respond to jasmonate applications with increased defense compounds (Farmer and Ryan 1992; Thaler et al. 1996; Lindroth and Kinney 1998; Cooper and Rieske 2008), which have negative consequences on herbivore growth, consumption, and population dynamics (Stout and Duffey 1996; Omer et al. 2000; Van Dam et al. 2000; Thaler et al. 2001; Gols et al. 2003; Cooper et al. 2004; Van Dam et al. 2004; Cooper et al. 2005; Cooper and Rieske 2008). Jasmonate applications also have antixenotic effects on feeding preferences and oviposition site selection of certain herbivores (Stout and Duffey 1996; Bruinsma et al. 2007; van Dam 2008) and increase airborne volatiles, which attract natural enemies (Thaler et al. 2001; Bruinsma et al. 2009) and pollinators (Radhika et al. 2010). In addition to their role in induction of plant defenses, jasmonates are involved in the activation of nutritive sinks (Creelman and Mullet 1997; Anrold and Schultz 2002; Meuriot et al. 2004; Babst et al. 2005) by regulating certain genes that encode vegetative storage proteins (Anderson 1991; Meuriot et al. 2004) and that are involved in tuber formation (Pelacho and Mingo—Castel 1991; Koda 1992; Staswick 1994). Treatment of *Medicago sativa* with methyl jasmonate increases nitrogen partitioning to vegetative sinks (Meuriot et al. 2004). In *Populus* spp., JA application induces rapid carbon export from both local and systemic leaves, and increases both carbon partitioning to vegetative sinks (Babst et al. 2005) and cell wall invertase activity associated with sink—strength (Arnold and Schultz 2002).

Given the role of jasmonates in plant defense and activation of nutritive sinks, it is conceivable that induction of jasmonates in plant tissues may influence aspects of insect—induced gall development and maintenance; particularly gall defenses and sink—strength. The objective of this study was to investigate the ecological consequences of exogenous applications of JA and a JA—inhibitor, diethyldithiocarbamic acid (DIECA), on the growth and defense of *D. kuriphilus* galls formed on American chestnut, *Castanea dentata* (Marsh.) Borkhausen (Fagales: Fagaceae), and Chinese chestnut, *C. mollissima* Blume; elucidating the physiological role of JA in gall formation and gall defenses was not a study objective. DIECA rapidly and efficiently causes the chemical reduction of hydroperoxyoctadecatrienoic acid, the product of the oxidation of linolenic acid by lipoxygenase, which prevents its cyclization and subsequent conversion to downstream products, including JA (Farmer et al. 1994). DIECA has been successfully used as a JA— inhibitor on a wide range of plant species (Piel et al. 1997; Gantet et al. 1998; Lee et al. 1998; Menke et al. 1999; Watanabe et al. 2000; Garcia—Ponce and Rocha—Sosa 2000; Bulgakov et al. 2004; Saltveit et al. 2005; Hu and Zhong 2008; Lee et al. 2008; Peebles et al. 2009). Elucidation of how JA manipulation influences gall characteristics could improve the interpretation of studies that investigate gall ecology.

## Materials and Methods

### Plant material

Heavily galled American and Chinese chestnut trees located at the American Chestnut Foundation Breeding Farm in Meadowview, VA were arbitrarily selected in early spring 2005 and 2006. Trees at this site were planted in rows of even—aged blocks in 1991 for the chestnut blight resistance breeding program conducted by the American Chestnut Foundation. Trees used in this study were ∼ 3–5 m in height. The gall wasp population was first observed in 2001, and was associated with large populations of parasitoids and chestnut weevil (Cooper and Rieske 2007, 2009, 2010, 2011).

### Treatment applications

Jasmonic acid (Sigma—Aldrich, www.sigmaaldrich.com) was dissolved in acetone at a concentration of 1 g/mL and dispersed in water to achieve a 1.5 m*M* solution (Thaler 1999; Cooper et al. 2004; Cooper and Rieske 2008). This concentration did not cause senescence or wilting on chestnut in a previous study (Cooper and Rieske 2008). An equal quantity of acetone without JA was dispersed in water for control treatments. Gall source leaves (2–6 leaves per gall) directly attached to galls (Cooper and Rieske 2009) on American (n = 36, 18 per treatment) and Chinese (n =16, 8 per treatment) chestnut trees were treated weekly at a rate of ∼ 1 mL per gall using an atomizer. Treatments began on 21 March 2005 and continued until 1 June 2005 for American chestnut and 25 May 2005 for Chinese chestnut, based on differences in tree phenology between these two chestnut species (Hebard 1994). The timing of treatment applications began prior to bud break and gall development, and ended prior to *D. kuriphilus* emergence but after the flight and oviposition period of the dominant parasitoid, *Torymus sinensis*, and after the emergence of the chestnut weevil from overwintering locations. Chestnut galls were collected from American (n = 128, 64 per treatment) and Chinese *(*n = 68, 34 per treatment) chestnuts on 1 June and 7 June 2005, respectively, for assessment of physical characteristics. Galls were kept on ice during transport and stored at -20 °C. Additional American (n = 24, 12 per treatment) and Chinese (n = 20, 10 per treatment) galls were collected concurrently for estimation of tannin contents; samples were frozen in liquid nitrogen and kept on dry ice during transport, and stored at -80 °C.

DIECA (Sigma—Aldrich) was prepared by dissolving it in water to make a 50 m*M* solution, and water without DIECA was used as a control (Lee et al. 1998). Gall source leaves (leaves directly attached to galls) on American (n = 8, 4 per treatment) and Chinese (n = 12, 6 per treatment) chestnut trees were treated with DIECA or control solution after lightly damaging gall leaves with forceps (Lee et al. 1998); damage by forceps was applied only on the first treatment date. DIECA treatments were applied weekly to source leaves at a rate of ∼ 1 mL per gall from 3 May through 18 May 2006 on American chestnut, and 18 April through 11 May 2006 on Chinese chestnut. For assessment of physical characteristics, American chestnut galls (n = 19 DIECA, n = 16 control) were collected on 25 May and Chinese chestnut galls (n = 32 DIECA, n = 28 control) were collected on 18 May. Additional American (n = 30, 15 per treatment) and Chinese (n = 30, 15 per treatment) chestnut galls were collected for estimation of tannin contents.

### Gall assessments

Gall volume was calculated using the equation for ellipse volume, ((4/3) × π × radius 1 × radius 2 × radius 3), where radii 1–3 were measured with calipers on three different planes across the gall (length and two right— angle planes for width) (Cooper and Rieske 2009, 2010). The gall exteriors were assessed for fungal lesions and weevil feeding punctures. Each gall was then dissected at 160× magnification to count the number of chambers that contained gall wasp larvae or parasitoids, or chambers that were empty (Cooper and Rieske 2009, 2010). Gall chambers were cross—sectioned and the diameter of sclerenchyma layers surrounding the chambers were measured using a micrometer (Cooper and Rieske 2009, 2010). Following dissections, galls were oven dried and the dry weight per number of chambers was recorded as a measure of nutritive value (Kato and Hijii 1993).

For tannin estimation, galls were crushed in liquid nitrogen with a mortar and pestle and lyophilized for 96 hours in a VirTis freeze dryer (SP Scientific, www.spscientific.com). Total tannins were estimated by a radial diffusion protein precipitation assay using tannic acid as a standard (Sigma—Aldrich lot # 107H1165) and bovine serum albumin as protein template (Hagerman 1987).

### Statistical analyses

Data were examined for heterogeneity of variance and non—normality of errors by inspecting residual and normal quantile—quantile plots, respectively. Based on plots, the proportion of chambers per gall that contained gall wasps, parasitoids, or that were empty were arcsine—square root transformed prior to analysis (Zar 1996). Analyses of the number of chambers per gall, number of weevil herbivory punctures per gall, and tannin contents were conducted using PROC GLIMMIX (SAS version 9.2) with chestnut species (American vs. Chinese), foliar applications (JA or DIECA vs. control), and their interactions as main effects. Analyses of gall characteristics (dry weight per chamber, gall volume, sclerenchyma layer thickness) and gall inhabitants (proportion of chambers that contained gall wasps, parasitoids, or that were empty) were conducted using PROC GLIMMIX (SAS version 9.2) with chestnut species (American vs. Chinese chestnut), foliar applications (JA or DIECA vs control), and their interactions as independent variables, and the number of chambers per gall as a covariate. When significant interactions between chestnut species and foliar applications were detected, means were compared using the ADJUST=SIMULATE option of the LSMEANS statement. Logistic regression (PROC LOGISTIC) was used to analyze the incidence of fungal lesions between chestnut species and treatment applications. For all statistical analyses, values were considered significantly different at α = 0.05.

## Results

### Ecological consequences of JA application

The number of chambers per gall did not vary between chestnut species (*F* = 2.2; df = 1, 193; *p* = 0.14) or foliar treatments (*F* = 0.1; df = 1, 193; *p* = 0.82), and there was no chestnut species by treatment application interaction (*F* = 0.18; df = 1, 193; *p* = 0.67). On average, both JA—treated and control galls contained 2.0 ± 0.2 (SE) chambers.

Analyses of gall characteristics detected significant chestnut species by foliar treatment interactions for gall dry weight (*F* = 10.1; df = 1, 191; *p* < 0.01), gall volume (*F* = 9.3; df = 1, 192; *p* < 0.01), the thickness of the sclerenchyma layer surrounding gall chambers (*F* = 8.6; df = 1, 192, *p* < 0.01), and the occurrence of external fungal lesions (χ^2^ = 5.9; df = 1; *p* < 0.05). The observed main effect interactions indicate the effects of JA treatment on these variables were not consistent between chestnut species. On Chinese chestnut, galls that were treated with JA had greater dry weight and volume, thicker sclerenchyma layers, and lower incidence of lesion infection compared with galls treated with controls. The same differences were not observed on American chestnut galls treated with JA and control (Table 1).

There was no significant chestnut species by foliar treatment interaction (*F* = 3.2; df = 1, 192; *p* = 0.08) or chestnut species effect (*F* = 0.4; df = 1, 192; *p* = 0.53) with respect to the proportion of chambers that were empty. However, galls that were treated with JA had a significantly lower proportion of empty chambers compared with control galls, regardless of chestnut species (*F* = 8.7; df = 1, 192; *p* < 0.01) (Table 2). There was no chestnut species by foliar treatment interaction (*F* = 0.2; df = 1, 18; *p* = 0.64) with respect to tannin content, though there were significant differences between foliar applications (*F* = 5.6; df = 1, 18; *p* < 0.05). Tannin estimates were higher in galls that were treated with JA compared with control, regardless of chestnut species (Table 2). There were also higher tannin levels in American chestnut galls (0.06 ± 0.005 mg tannic acid equivalent/mg dry tissue wt) compared with Chinese chestnut galls (0.031 ± 0.006 mg tannic acid equivalent/mg dry tissue wt), regardless of foliar applications (*F* = 13.7; df = 1, 18; *p* < 0.01).

Analysis of the number of weevil feeding puncture wounds per gall did not reveal a significant chestnut species by foliar application interaction (*F* = 2.0; df = 1, 192; *p* = 0.16) or significant differences between chestnut species (*F* = 0.01; df = 1, 192; *p* = 0.98) or foliar application (*F* = 0.12; df = 1, 192; *p* = 0.73).

### Ecological consequences of DIECA application

The number of chambers per gall did not vary between chestnut species (*F* = 3.5; df = 1, 91; *p* = 0.07) or treatment (*F*= 0.5; df = 1, 91; *p* = 0.49), and there was no chestnut species by treatment application interaction (*F* = 2.7; df = 1, 91; *p* = 0.13). On average, both DIECA— treated and control galls contained 2.3 ± 0.2 (SE) chambers.

There were significant chestnut species by foliar application interactions with respect to gall dry weight (*F* = 3.9; df = 1, 89; *p* < 0.05) and the number of weevil feeding punctures per gall (*F* = 9.7; df = 1, 90; *p* < 0.01). American chestnut galls that were treated with DIECA had lower dry weights and more weevil feeding punctures compared to American chestnut galls treated with deionized water control. The same differences were not observed between Chinese chestnut galls treated with DIECA and control (Table 3).

Analysis of gall volume did not detect a significant chestnut species by foliar treatment interaction (*F* = 2.4; df = 1, 89; *p* = 0.13) or a significant difference between chestnut species (*F* = 0.28; df = 1, 89; *p* = 0.60), but the size of galls that were treated with DIECA were significantly smaller compared to galls that were treated with water controls, regardless of chestnut species (*F* = 5.2; df = 1, 89; *p* = 0.03) (Table 4). Logistic regression of the occurrence of fungal lesions on gall exteriors did not detect a significant chestnut species by foliar application interaction (χ*2* = 0.3; df = 1; *p* = 0.60) or significant differences between chestnut species (χ^2^ = 0.9; df = 1; *p* = 0.35). However, DIECA treatment increased the incidence of fungal lesions on both American and Chinese chestnut galls (χ^2^ = 3.9; df = 1; *p* < 0.05) (Table 4).

Analysis of the proportion of empty chambers per gall did not reveal significant differences among chestnut species (*F* = 2.3; df = 1, 90; *p* = 0.13), foliar application (*F* = 1.5; df = 1, 90; *p* = 0.22), or their interaction (*F* = 3.7; df = 1, 90; *p* = 0.06). Analysis of sclerenchyma layer thickness did not reveal a significant main effect interaction (*F* = 1.6; df = 1, 90; p = 0.22) or a significant treatment effect (*F* = 0.19; df = 1, 90; *p* = 0.67). However, sclerenchyma layers within Chinese chestnut galls were significantly thicker (5.7 ± 0.3 mm) than those within American chestnut galls (3.3 ± 0.4 mm) (*F* = 18.1; df = 1, 90; *p* < 0.01). Analysis of gall tannins did not reveal a significant interaction between main effects (*F* = 0.2; df = 1, 59; *p* = 0.69) or a significant effect for foliar application (*F* = 1.6; df = 1, 59; *p* = 0.21). However, American chestnut galls had higher tannin content (0.095 ± 0.011 mg tannic acid equivalent/mg dry tissue wt) compared with Chinese chestnut galls (0.044 ± 0.011 mg tannic acid equivalent/mg dry tissue wt) regardless of foliar treatment (*F* = 13.5; df = 1, 59; *p* < 0.01).

### Effects of chestnut species on *Dryocosmus kuriphilus* gall inhabitants

The analyses of the proportion of gall chambers that contained gall wasps did not reveal chestnut species by foliar treatment interactions in either 2005 (*F* = 0.16; df = 1, 192; *p* = 0.69) or 2006 (*F* = 0.06; df = 1, 90; *p* = 0.16), and also showed no significant foliar treatment effects for JA in 2005 (*F* = 0.4; df = 1, 192; *p* = 0.53) or DIECA in 2006 (*F* = 0.3; df = 1, 90; *p* = 0.58). Significant differences were found, however, between chestnut species in both 2005 (*F* = 33.5; df = 1, 192; *p* < 0.01) and 2006 (*F* = 28.8; df = 1, 90; *p* < 0.01). More chambers within American chestnut galls contained gall wasp larvae when compared with Chinese chestnut galls in both study years (Table 5).

Analyses of the proportion of gall chambers that contained parasitoids did not reveal significant chestnut species by foliar treatment interactions in either 2005 (*F* = 0.69; df = 1, 192; *p* = 0.41) or 2006 (*F* = 2.6; df = 1, 90; *p* = 0.11). No significant differences were found among foliar treatment effects for JA in 2005 (*F* = 1.9; df = 1, 192; *p* = 0.17) or DIECA in 2006 (*F* = 0.38; df = 1, 90; *p* = 0.54), though there was a significant difference between chestnut species in both 2005 (*F* = 19.4; df = 1, 192; *p* < 0.01) and 2006 (*F* = 9.1; df = 1, 90; *p* < 0.01). Fewer chambers within American chestnut galls contained parasitoids when compared with Chinese chestnut galls (Table 5).

## Discussion

The patterns observed in this study suggest that JA application to source leaves of *D. kuriphilus* galls increase gall size and defense, and that effects of JA application were greater for galls on Chinese chestnut compared with galls on American chestnut. JA treatment of source leaves of *D. kuriphilus* galls on Chinese chestnut increased gall dry weight, gall volume, and sclerenchyma layer thickness, and decreased the incidence of fungal lesions on the gall exteriors. However, JA treatment did not have the same effects on galls from American chestnut. Treatment of gall source leaves with DIECA, which inhibits JA synthesis, decreased gall dry weight and increased weevil herbivory on American chestnut galls, but not on galls from Chinese chestnut. One potential explanation for the species—specific responses to JA and its inhibitor may be found in the context of species—specific responses to infection by the chestnut blight fungus. The blight fungus causes cankers on susceptible chestnut stems by destroying vascular tissues (Hebard et al. 1984), and JA synthesis increases following tissue damage and water stress (reviewed by Creelman and Mullet 1997; Schilmiller and Howe 2005). The American chestnut is highly susceptible to the blight fungus, and those used in this study were infected. Consequently, endogenous JA levels may have been high in the American chestnut trees, and therefore difficult to increase using exogenous applications. In comparison, endogenous JA levels in the Chinese chestnut trees, which are resistant to the blight fungus, may have been difficult to influence using the JA inhibitor. These observations warrant further research.

JA and DIECA treatments influenced galls and gall wasps, and the overall effects of DIECA treatments were generally opposite from the effects of JA treatments. Treating chestnut gall leaves with JA reduced the occurrence of empty gall chambers in both chestnut species. Empty chambers represent early mortality of gall wasp larvae, and are thought to be caused by low host quality, host plant resistance, and/or parasitoids (Otake 1989; Kato and Hijii 1999; Cooper and Rieske 2009, 2010). The changes in the frequency of empty chambers may be explained by modifications in gall characteristics relevant to gall inducer defense and nutrition. JA treatment increased the dry weight, volume, and sclerenchyma thickness of Chinese chestnut galls, whereas DIECA decreased the dry weight of American chestnut galls and the volume of galls from both chestnut species. Sclerenchyma layer thickness and gall volume may provide physical defenses that provide gall wasp larvae with protection from parasitoid oviposition and/or other mortality factors (Stone et al. 2002; Cooper and Rieske 2010). Kato and Hijii (1993) reported that the dry weight of galls is positively correlated with *D. kuriphilus* fecundity, and was used as a measure of nutritive value in their study. JA has been shown to increase cell wall invertases, which are enzymes responsible for sink strength, and increase carbon partitioning to vegetative sinks (Arnold and Schultz 2002; Babst et al. 2005). Potentially, JA may increase the sink—strength of developing galls, which may increase survivorship of gall inhabitants.

JA treatment of gall source leaves also influenced gall defenses, which may have contributed to the reduction of empty chambers. JA treatment decreased the incidence of external fungal lesions on Chinese chestnut galls and increased gall tannins on both chestnut species, while DIECA treatment increased the incidence of external fungal lesions on galls from both chestnut species. The lesion—causing fungus is currently unidentified, but similar lesions are formed on oak galls by fungal endophytes (Butin 1992; Wilson 1995), and can contribute to gall wasp mortality (Carroll 1995; Wilson 1995; Cooper and Rieske 2010). Fungal endophyte infections are suppressed by high tannin content on oak (Taper and Case 1987). Lesion formation on chestnut galls may have been reduced by JA induced tannins (Cooper and Rieske 2008), but there was no clear correlation between tannin estimates and lesion formation. However, chestnuts demonstrate species—specific differences in tannin composition (Cooper and Rieske 2008), which may have influenced tannin estimates. DIECA treatment also increased the incidence of herbivory by the lesser chestnut weevil on American chestnut galls, perhaps due to lowered gall defenses or increased phagostimulants not tested for this study.

Parasitism rates were higher and the presence of gall wasp larvae lower in galls on Chinese chestnut compared with galls on American chestnut in both years. In fact, the percentage of gall chambers within Chinese chestnut galls that contained gall wasps was under 10% in both years. Comparatively, the percentage of chambers within American chestnut galls that contained gall wasps was over 30% in 2005, and reached nearly 50% in 2006. These patterns suggest that parasitism could suppress gall wasp populations on Chinese chestnut trees at this location, but low parasitism rates in the American chestnut galls provide a source population for continuous re-colonization of Chinese chestnut trees.

Differences in tree phenology between American and Chinese chestnuts may increase the susceptibility of Chinese chestnut galls to parasitism. Chinese chestnut budburst and leaf expansion occurs ∼ two weeks prior to American chestnut at this location (36° 45′ N, 81° 51′ W) (Hebard 1994), and the formation of *D. kuriphilus* galls follows the same trend (WRC personal observation). The dominant parasitoid of *D. kuriphilus* is the introduced *T. sinensis* (Cooper and Rieske 2007, 2011), which emerges from overwintering sites in early spring, concurrent with new gall development. *T. sinensis* was introduced from Asia (Payne 1978; Cooper and Rieske 2007) and has a long evolutionary history with Chinese chestnut and *D. kuriphilus.* The timing of emergence of *T. sinensis* from their overwintering locations in dead galls is synchronized with the development of new galls on Chinese chestnut. Potentially, the later gall formation on American chestnut provides a mechanism for escape—in—time from *T. sinensis.* Similarly, oak phenology also affects parasitism rates on another cynipid wasp, *Aphelonyx glanduliferae* (Ito and Hijii 2002). The novel associations of *D. kuriphilus* and *T. sinensis* on American chestnut warrant long—term studies to assess potential changes in the timing of *T. sinensis* emergence with respect to selective pressures from American chestnut phenology, and potential changes in the frequency of *T. sinensis* parasitism in American chestnut galls.

This study is the first to evaluate the affects of JA treatment on insect galls, and suggests that JA induction could benefit gall wasps by increasing gall size and defense, and reducing the mortality agent(s) that cause empty chambers. Factors which induce JA synthesis in host plants—such as foliar herbivory— could alter gall development and defense. Additionally, phenological asynchrony between the gall—maker and its novel American chestnut host may decrease the susceptibility of *D. kuriphilus* to parasitism by the non—native parasitoid *T. sinensis.* Potentially, cultivated chestnuts selected for early bud—break and leaf expansion concurrent with parasitoid emergence from overwintering sites could provide chestnut cultivars that promote parasitism.

**Table 1.**
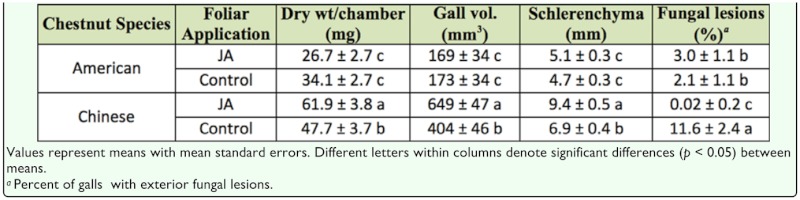
Effects of chestnut species and JA application on Dryocosmus *kuriphilus* gall characteristics and lesion formation.

**Table 2.**
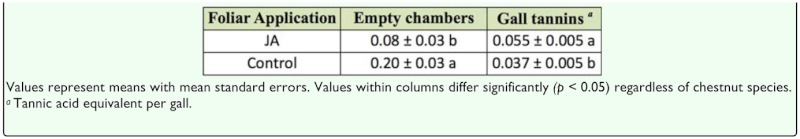
Effects of exogenous JA application on the proportion of *Dryocosmus kuriphilus* gall chambers that were empty and on estimates of gall tannins.

**Table 3.**
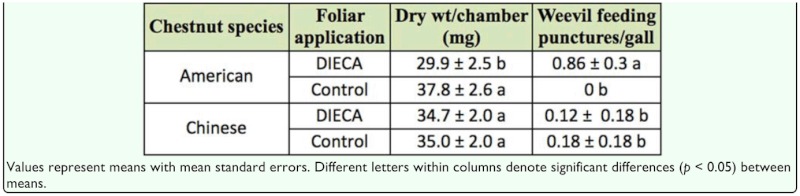
Effects of chestnut species and application of a JA inhibitor (DIECA) on *Dryocosmus kuriphilus* gall dry weight and the number of weevil feeding punctures per gall.

**Table 4.**
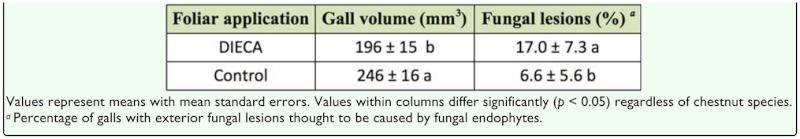
Effects of a JA inhibitor (DIECA) (2006) on gall volume and occurrence of fungal lesions.

**Table 5.**
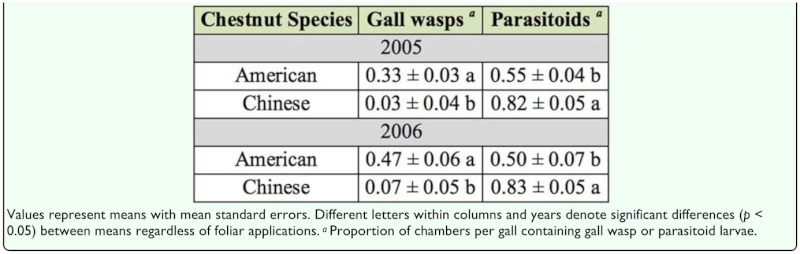
Effects of chestnut species on *Dryocosmus kuriphilus* gall inhabitants in 2005 and 2006.

## Abbreviations:

**JA**,: jasmonic acid;
**DIECA**,: diethyldithiocarbamic acid
